# Direct and indirect associations among mothers’ invalidating childhood environment, emotion regulation difficulties, and parental apology

**DOI:** 10.1186/s40479-022-00191-z

**Published:** 2022-08-18

**Authors:** Alexis A. Adams-Clark, Angela H. Lee, Yoel Everett, Arianna Zarosinski, Christina Gamache Martin, Maureen Zalewski

**Affiliations:** 1 Department of Psychology, University of Oregon, 1227 University St., Eugene, OR 97401 USA; 2 Private Practice, Eugene, OR USA

**Keywords:** Apology, Parenting, Invalidating environment, Emotion regulation

## Abstract

**Background:**

Effective emotion regulation abilities are essential for engaging in positive, validating parenting practices. Yet, many parents report difficulties with both emotion regulation and positive parenting, and these difficulties may in part be the result of parents’ own childhood experiences of invalidation. Building upon prior literature documenting the intergenerational transmission of invalidation and emotion dysregulation, the present study examined the associations between these constructs and a specific parenting practice – parental apology – that can be conceptualized as a type of validating parenting practice.

**Methods:**

Using a sample of 186 community mothers, we tested direct and indirect relationships via correlational and path analysis between participants’ retrospective reports of parental invalidation during childhood, difficulties with emotion regulation, and two aspects of parental apology – proclivity (i.e., participants’ self-reported propensity to apologize to their child) and effectiveness (i.e., participants’ inclusion of specific apology content when prompted to write a child-directed apology). Parental invalidation, difficulties with emotion regulation, and parental apology proclivity were measured via self-report questionnaires. Apology effectiveness was measured by coding written responses to a hypothetical vignette.

**Results:**

There was a significant negative bivariate relationship between difficulties with emotion regulation and parental apology proclivity and effectiveness. Parents’ own childhood experiences of invalidation were linked to parental apology indirectly via emotion regulation difficulties.

**Conclusions:**

Results suggest that mothers with greater difficulties regulating emotions may be less able to or have a lower proclivity to apologize to their child when appropriate. Thus, parent apology may be an important addition to current calls for parent validation training.

**Supplementary Information:**

The online version contains supplementary material available at 10.1186/s40479-022-00191-z.

## Background

Parenting is inherently stressful and emotionally evocative, making parents’ ability to regulate their emotions an essential component of effective parenting [1]. Effective emotion regulation is important, yet particularly challenging, during emotionally laden parenting situations, such as when a parent makes a mistake that warrants an apology (e.g., blaming child for something the child did not do). Although there has been a recent call to increase attention to emotion regulation as the most proximal contributor to many parenting behaviors [2], studies examining the associations between parents’ emotion regulation and parental apology are lacking. Moreover, little is known regarding determinants of individual differences in parents’ emotion regulation capacities, though one potential link is parents’ own experiences of having their emotions invalidated during childhood [3]. Therefore, in this study, we examined the associations between parents’ emotion regulation difficulties, parents’ own invalidating environments during childhood, and parental apology. Further, we examined the indirect role of emotion regulation difficulties in linking parents’ invalidating childhood experiences to parental apology behaviors.

### Invalidation and the development of emotion regulation difficulties

Two distinct yet converging literatures provide support for the idea that emotion regulation difficulties develop from invalidating parenting environments and are, in turn, prone to intergenerational transmission. The first literature comes from the field of child development and examines the role of emotion socialization. Parental emotion socialization refers to the manner in which parents respond to their own and their children’s emotions [4]. Specifically, emotion socialization theories posit that parent emotion regulation is related to the development of children’s emotion regulation through parental modeling of effective emotion regulation, as well as through supportive responses to children’s emotions [5]. Parents who can more effectively regulate their emotions in an emotionally laden parenting context may respond to their children’s emotions in supportive and validating ways. For example, Are and Shaffer [6] found that parents with fewer emotion regulation difficulties were more likely to endorse an environment of positive emotion expressiveness in the family, which subsequently predicted more effective child emotion regulation.

The second literature on the development of emotion regulation difficulties comes from the field of clinical intervention and similarly examines the developmental impacts of an invalidating environment. The biosocial model [7] suggests that a chronic invalidating environment in childhood accounts for individual differences in the development of emotion dysregulation. Such invalidating environments are characterized by features such as repeatedly communicating to someone that their experiences and feelings are wrong and inaccurate; minimizing difficulties; and discouraging the expression of negative emotions [7, 8]. Parents play a primary role in shaping children’s emotional environment, and chronic parental invalidation of children’s emotions is linked to subsequent difficulties with emotion regulation [9, 10].

Together, these distinct literatures converge to provide compelling support for the idea that invalidating emotional environments in childhood confer risk for the development of difficulties with emotion regulation and suggest a pathway for the intergenerational transmission of parental invalidation through emotion regulation difficulties. In support of this pathway, results of a recent study indicated mothers and fathers who report experiencing invalidation in their own childhood engage in more invalidation of their children’s emotions, and this association was mediated by parents’ emotion regulation deficits [11]. Other research suggests that such parenting behaviors are related to children’s emotion dysregulation [3]. Thus, emotion dysregulation may be both a cause and a mechanism of parental invalidation.

### Parental apology as validation

One understudied parenting behavior related to validation and invalidation is parents’ proclivity and ability to apologize to their children after a parenting mistake. In general, an apology is a complex speech act with important social implications. An apology is commonly provided in situations in which an individual commits a transgression or violation against another [12]. Because of its complexity, apology has been studied from a variety of theoretical perspectives. Generally, research on apology in psychology has examined an individual's tendency or *proclivity* to apologize in a situation in which they committed a transgression [13] and the effectiveness of their apology, as assessed by examining whether the necessary components of an apology are included in the apology [14]. Apology proclivity and apology effectiveness are both necessary when considering apology, as even individuals who recognize their wrongdoing and apologize may not include the elements of apology that facilitate healing and repair.

Given that parents do make mistakes, the parent–child relationship is rife for opportunities to apologize. Parental apology represents a validating parenting practice, as the admission of wrongdoing and expression of remorse communicate that the child’s experience and feelings resulting from the parental transgression are warranted [15]. In contrast, a failure to apologize, or engaging in ineffective apology, may represent a form of parental invalidation that communicates that a child’s understanding of their own experiences and emotions is inaccurate and wrong. When parents fail to repeatedly apologize, this may constitute chronic invalidation.

Although parental apology is of increasing interest in the media [16], little empirical research has been conducted with only a few notable exceptions [17, 18]. Both aspects of apology – proclivity and effectiveness – have been examined in this preliminary literature base. Ruckstaetter and colleagues [18] examined individual differences in parents’ proclivities to apologize to their child. Findings suggested that proclivity to apologize is an important measure of interest in the context of parenting, as it was related to greater parental empathy and to more secure parent–child attachment [18]. Adams-Clark and colleagues expanded upon this research by examining how mothers’ apology proclivity was related to apology effectiveness. In this study, the content of parental apologies was coded for specific components that have been established as particularly important for forgiveness and relationship repair in the general apology literature, such as expression of remorse, acknowledgement of responsibility, offer of repair, and promise of forbearance [19]. Results indicated that there was only a small, positive correlation between apology proclivity and apology effectiveness. Although mothers recognized their parenting mistake and offered an apology to their child, making an apology did not automatically equate to making an effective apology, suggesting a need to better understand factors that may make it more challenging to effectively apologize.

### Emotion regulation difficulties and apology

While effective emotion regulation is critical to engaging in effective and validating parenting behaviors generally, it may be particularly necessary for apologies. Apologies often occur in highly emotional contexts of interpersonal conflict [20]. Such emotionally charged situations can elicit threatening and aversive emotional experiences that impact one’s proclivity and ability to effectively apologize [21]. For example, previous research suggests that higher levels of shame may impede apology [22]. Similarly, mindfulness, which has been robustly associated with higher levels of emotion regulation, has been associated with higher trait and state levels of apology [23]. Thus, in the context of parenting mistakes, parents may need to regulate their own emotions to engage in more effective apologies, and parents with greater difficulties with emotion regulation may engage in less effective apology-specific behaviors.

### The current study

Previous research provides support for the intergenerational transmission of parental invalidation [11] and its links to emotion regulation difficulties [3], but no study has examined these relationships with regard to parental apology. The current study sought to fill this gap by examining the pathways between mothers’ childhood experiences of invalidation, emotion regulation difficulties, and two facets of maternal apology – mothers’ proclivity to apologize and their apology effectiveness. Mothers’ histories of being invalidated in childhood may have both a direct and indirect influence on parental apology behaviors. As described above, having experienced an invalidating environment during childhood may indirectly influence parents’ own apology behaviors via emotion regulation, such that invalidating environments lead to subsequent emotion regulation difficulties, which makes it harder to apologize effectively. Invalidating environments may additionally have a direct influence on apology, such that individuals who did not have parents demonstrating validating parenting behaviors presumably had fewer apologies modeled and may have a lower proclivity to apologize to their children or have less skill in crafting an effective apology. Specifically, we hypothesized that:

1. Mothers’ self-reported emotion regulation difficulties would be negatively associated with their apology proclivity and apology effectiveness.
2. Mothers’ self-reported childhood experiences of invalidation would be negatively associated with their apology proclivity and apology effectiveness.
3. Mothers’ self-reported childhood experiences of invalidation would be indirectly associated with their apology proclivity and apology effectiveness through emotion regulation difficulties.

## Methods

### Participants

Participants were 186 mothers (*M*_age_ = 33.17 years, *SD* = 4.83) with at least one child between the ages of 7 and 12 (*M*_childage_ = 8.95 years; *SD* = 1.61; 55.4% male, 43.5% female, 1.1% gender not listed). The majority of participants identified as White (93.2%), Non-Hispanic/Latino (94.4%), and heterosexual/straight (73.4%). Participants were recruited using a research database maintained by the psychology department of a public university in the northwest United States, which contains contact information for local families who have consented to be contacted about opportunities to participate in developmental psychology research (see https://teamduckling.uoregon.edu/ for more information). Additional eligibility criteria to participate in the study included being at least 18 years of age, identifying as a woman, and being a primary caregiver of a child. Recruitment was stratified based on income, such that study participants fell within one of three household income brackets: 1) below $35,000 (24.7%), 2) $35,000-$75,000 (38.2%), and 3) above $75,000 (37.1%). The recruitment process for this study and sample demographics are described further in [17].

### Procedure

Interested participants completed a screening survey to determine eligibility. Eligible participants were directed to the study survey, which was created using Qualtrics survey software. Participants completed the survey using an electronic device at their preferred time and location. The survey took approximately one hour to complete. Participants were compensated with a $15 Amazon gift card. Informed consent was obtained from all participants, and study procedures were approved by the affiliated university’s Institutional Review Board.

### Measures

#### Maternal proclivity to apologize

Mothers’ proclivity to apologize to their child was measured using the Proclivity to Apologize Measure-Parent (PAM-P; [18]). The PAM-P was created by adapting the pre-existing, general Proclivity to Apologize measure (PAM; [13]). In the adaptation, Ruckstaetter and colleagues [18] revised the original items to apply to apology proclivity in the context of the parent–child relationship. The PAM-P consists of eight negatively valanced statements (e.g., “I don’t like to admit to my child that I am wrong”). One additional question with a positive valance (“I have a tendency to apologize to my child”) was added to the measure, consistent with prior research on the PAM [24], for a total of nine items. Participants were asked to indicate their agreement with each item using a 7-point Likert scale (1 = “strong disagreement,” 7 = “strong agreement”). The original eight negatively valanced PAM-P items were reverse coded such that higher values indicated a greater proclivity to apologize to their child. The nine PAM-P items were averaged to create an average PAM-P score for each participant. The PAM-P (*α* = 0.82) demonstrated excellent internal consistency in this study.

#### Maternal apology effectiveness

Maternal apology effectiveness was measured by coding mothers’ written responses to a fictional vignette, in which a mother becomes angry and yells at her child after falsely accusing them of failing to put their bicycle away. Participants were asked to read the vignette and script a verbatim apology for the scenario. This vignette has been previously described in [17] and is included in the supplementary materials*.*

### Coding scheme

Mothers’ scripted apologies were coded using a directed content analysis approach [25]. Vignette responses were coded for components identified as integral in the construction of an effective apology by prior theoretical and empirical apology research [14, 15, 26, 27]. Specifically, the presence or absence of the following six apology components were coded: 1) remorse/regret (e.g., “I’m sorry for yelling at you”), 2) acknowledgement of unjust or wrong events (e.g., “Yelling at you was wrong”), 3) acknowledgement of victim’s emotions and/or suffering (e.g., “I can see that you’re crying and that I hurt your feelings”), 4) commitment of forbearance (e.g., “I promise that I will work on this in the future”), 5) offer of repair (“What can I do to make you feel better right now?”), and 6) explanation/rationalization of what went wrong (e.g., “I yelled because I had a rough day and jumped to conclusions”). Based on prior literature suggesting that apologies with a greater number of apology elements are more effective [14], components were summed to create a total score reflecting apology effectiveness that was used in data analysis. The coding manual with examples can be found at: https://mfr.osf.io/render?url=https://osf.io/c36dy/?pid=w74cy%26direct%26mode=render%26action=download%26mode=render.

Vignettes were coded by two members of the study team. Interrater reliability was estimated based on a random sample of 33% (*n* = 63) of the files, using percent agreement and Cohen’s kappa statistic. The average Cohen’s kappa was 0.77, and the average percent agreement was 93.4% across the six components, indicating substantial agreement. However, examining interrater reliability for individual components revealed that the percent agreement and Cohen’s kappa were lower (76.2% and 0.37, respectively) for the “explanation/rationalization of what went wrong” category compared to the other dimensions, and the decision was made to exclude this component for further analyses. After removing this category, the average percent agreement for the five apology components used in subsequent analyses was 96.8% and Cohen’s kappa was 0.87, indicating near perfect agreement. Thus, the final categories included – remorse/regret, acknowledgement of unjust/wrong events, acknowledgement of harm/emotions, commitment of forbearance, and offer of repair. Coding discrepancies were resolved through discussion with the study team.

#### Difficulties with emotion regulation

Mothers’ difficulties with emotion regulation were measured using the Difficulties in Emotion Regulation Scale (DERS; [28]). The DERS is a 36-item self-report questionnaire in which mothers were asked to rate the frequency that each item relates to them on a 5-point Likert scale (1 = “almost never”, 5 = “almost always”). All items are summed to yield a total score, such that higher scores indicate greater difficulty with emotion regulation. The DERS also yields subscales related to emotional awareness, emotional clarity, regulation strategies, impulse control, goal-directed behavior, and nonacceptance of emotions. For this study, the DERS total score was used in primary analyses to gauge participants’ global emotion regulation difficulties. The DERS yielded good internal consistency (*α* = 0.96) in this study.

#### Childhood experiences of parental invalidation

The Invalidating Childhood Environment Scale (ICES; [29]) was used to retrospectively assess mothers’ childhood experiences of parental invalidation. The ICES is a retrospective self-report measure in which participants are asked to rate 14 parental behaviors towards them during childhood by both their mothers and fathers (e.g., “When I was anxious, my parents ignored this”) on a 5-point Likert scale (1 = “never”, 5 = “all the time”). Items are summed to create a total score, such that higher scores indicate higher levels of perceived parental invalidation. The ICES has been shown to demonstrate excellent internal consistency and construct validity samples [29]. In the current sample, the ICES yielded good internal consistency for participants’ mothers (*α* = 0.92) and fathers (*α* = 0.93). In this study, both mother and father scores were summed to create a total score for childhood experiences of parental invalidation, as both scores were highly correlated, *r* = 0.60, *p* < 0.001.

### Data analysis

#### Pre-Registered Analysis Plan

The hypotheses and analysis plan were pre-registered on the Open Science Framework (OSF) prior to data analysis (available at https://osf.io/8agx5/?view_only=62f618f673e84b8f88d9cf92e12ca4a9). To address our first and second study aim, we calculated descriptive statistics and Pearson *r *correlation coefficients. To test whether childhood experiences of invalidation were indirectly related to experiences of parent apology characteristics through difficulties with emotion regulation, we examined a path analysis model to estimate both the direct association of childhood experiences of invalidation on the two facets of apology, as well as indirect associations through emotion regulation difficulties. The model was tested with 10,000 bias-corrected bootstrapped samples.

#### Covariate inclusion

Covariate inclusion decisions were made based on the results of a previous study using the same sample [17]. This preliminary study was distinct in that it examined the measurement of apology effectiveness in detail, the relationship between apology effectiveness components, and their relationships with apology proclivity. Results from this study did not indicate any demographic differences in apology variables, with the exception of apology effectiveness by education *F*(3, 173) = 2.68, *p* = 0.05, such that higher education level was related to higher apology effectiveness scores. Differences were examined using age, education status, household income, race, ethnicity, relationship status, and sexual orientation. Based on these prior results, we ran the path model in this study with and without covarying for education level. This did not change statistical conclusions, so we opted to present the model statistics without the education variable for parsimony and consistency with pre-registered analysis plan.

#### Missing data

Rates of missing data were low (0% for apology variables; 3.8% for DERS; 13.4% for ICES). The higher rate of missing data for the ICES was likely due to its positioning at the end of the study, with participant fatigue contributing to dropout. Full Information Maximum Likelihood was used to handle missing data within path analyses. Data were examined for univariate outliers (defined as exceeding 1.5 multiplied by the interquartile range). Univariate outliers were capped at values corresponding to the lower or upper 5% of the respective distributions. However, these outlier procedures did not affect any statistical conclusions, so we opted to present the results from the raw data.

#### Statistical software

Data were analyzed using the *R* package *lavaan* (Version 0.6.7; [30])*.* Two-tailed statistical tests with a significance threshold of *p* < 0.05 were used.

## Results

### Bivariate relationships between ICES, DERS, and apology

Descriptive statistics were calculated for each variable and are listed in Table 1. Mothers’ difficulties with emotion regulation were negatively related to proclivity to apologize to their child, *r*(178) = -0.39, *p* < 0.001, and apology effectiveness, *r*(178) = -0.18, *p* = 0.019. However, mothers’ childhood invalidation was not related to apology proclivity, *r*(160) = -0.12, *p* = 0.126, or apology effectiveness, *r*(160) = 0.00, *p* = 0.951. Mothers’ apology proclivity and apology effectiveness were positively related, *r*(185) = 0.23, *p* = 0.001,^1^ and mothers’ difficulties with emotion regulation were positively related to their childhood experiences of invalidation, *r*(158) = 0.29, *p* < 0.001. In exploratory, follow-up analyses (not pre-registered), we examined the association of apology variables with DERS subscales. Apology proclivity was significantly associated with all DERS substances, all *p*’s < 0.01 (nonacceptance of emotions, goal-directed behavior, impulse control difficulties, lack of emotional awareness, limited emotion regulation strategies, and lack of emotional clarity). However, apology effectiveness was only significantly related to lack of emotional awareness, *p* = 0.001.

**Table 1:**
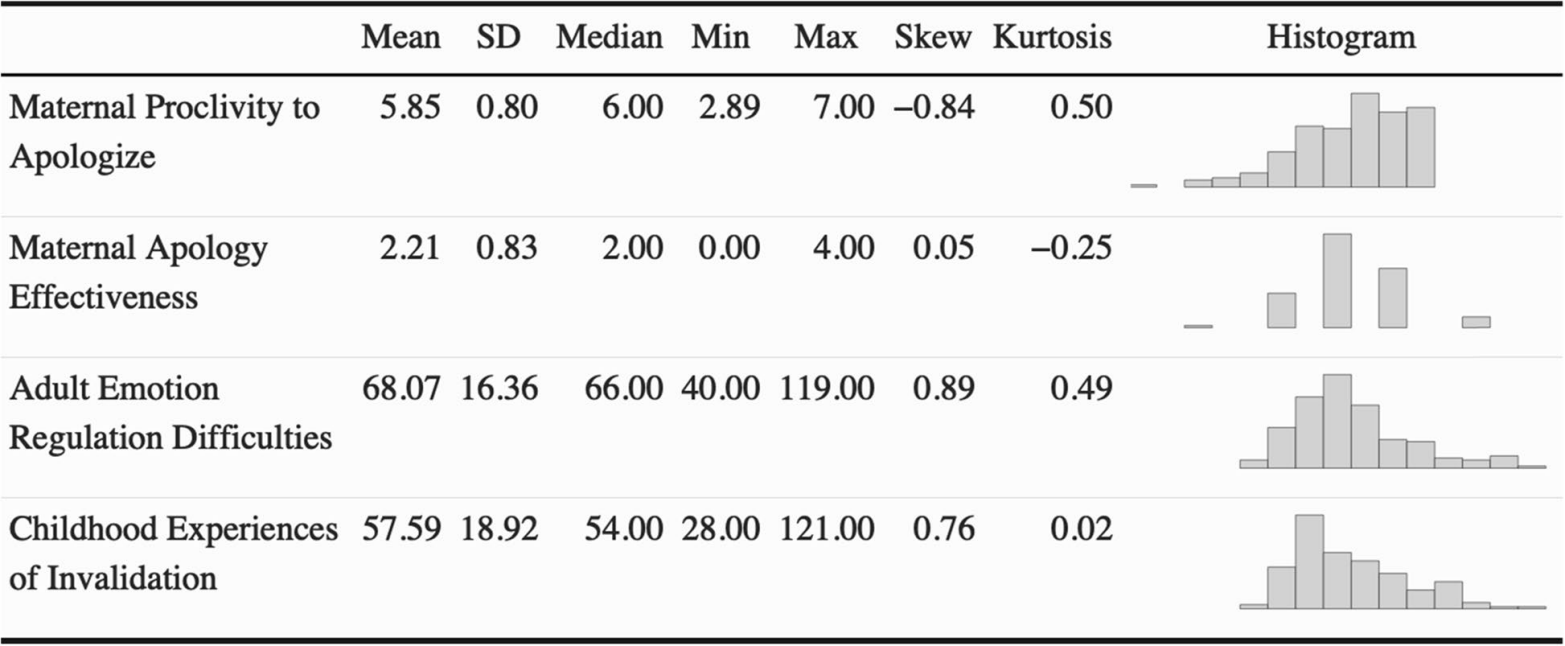
Descriptive Statistics for Continuous Variables (*N* = 186)

### Path model examining ICES, DERS, and apology

**Fig. 1:**
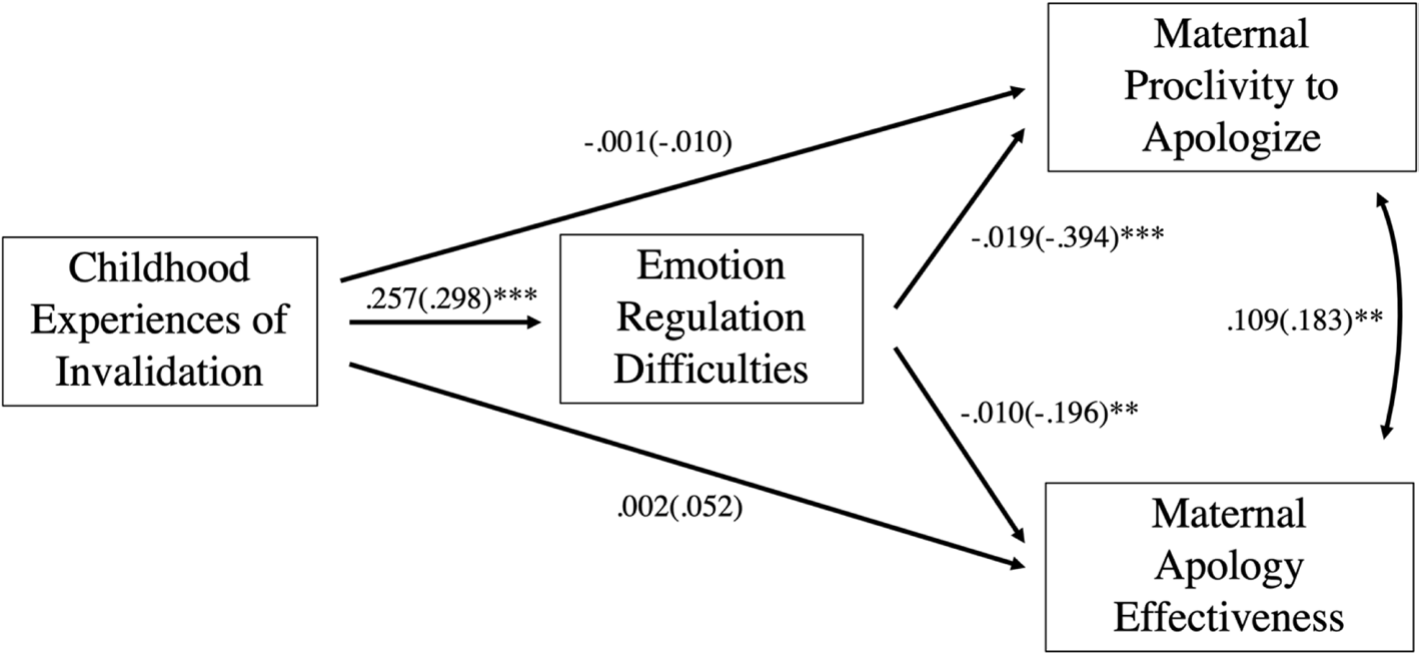
Unstandardized (and Standardized) Parameter Estimates for Path Model of Maternal Proclivity to Apologize and Maternal Apology Effectiveness (*N* = 186). Note. **p < .01, ***p < .001

Path analysis indicated a significant indirect effect of childhood experiences of invalidation on both apology proclivity and apology effectiveness through difficulties with emotion regulation. The bootstrapped unstandardized indirect effect for apology proclivity was estimated to be -0.005, *z* = -2.77, *p* = 0.006. The bootstrapped unstandardized indirect effect for apology effectiveness was estimated to be -0.003, *z* = -2.18, *p* = 0.029 (see Fig. 1). There were no significant direct or total effects of childhood experience of invalidation on apology proclivity or apology effectiveness.

## Discussion

Drawing on concepts from the complementary literatures on parenting, emotion regulation, and apology, the current study investigated direct and indirect relationships between mothers’ perceptions of parental invalidation during childhood, difficulties with emotion regulation, and use of apology within a parenting context. We hypothesized that mothers’ childhood experiences of invalidation would relate to their apology proclivity and effectiveness both directly and indirectly via difficulties with emotion regulation. Overall, our hypotheses were partially supported. As hypothesized, there were significant bivariate relationships between difficulties in emotion regulation and both parental apology variables, and parental invalidation experiences were linked to parental apology variables indirectly via their difficulties with emotion regulation. However, parental invalidation experiences were not directly related to parental apology.

In our path model, there was a significant positive relationship between mothers’ childhood experiences of parental invalidation and their own difficulties in emotion regulation. This relationship was expected and consistent with the biosocial theory [7], parental emotion socialization research [5], and prior research both within [10] and outside [31] the domain of parenting. The second component of our model, however, tested the novel associations between mothers’ emotion regulation and apology. Greater difficulties with emotion regulation were associated with a lower parental proclivity to apologize and a less effective apology in the hypothetical parenting vignette.

Although Ruckstaetter and colleagues [18] speculated about the necessity of emotion regulation capacities for parental apology, this study is the first study to our knowledge to empirically link overall emotion regulation capacities with any apology-related variable. Despite the lack of literature linking parental apology specifically to emotion regulation in any context, this relationship is consistent with patterns in the general parenting and emotion regulation literature that indicate that positive and validating parenting practices, akin to parental apology, often require parents to regulate emotions. In addition, it is also consistent with statements made in the apology literature. In general, scholars theorize that apology requires complex emotion regulation processes; to apologize, one must downregulate the emotion of shame that often emerges after committing a transgression against another person, while simultaneously acting on the emotion of guilt [22]. Such is a challenging feat for even those with well-developed emotion regulation capacities.

Providing mixed support for our other hypotheses, mothers’ childhood experiences of invalidation were indirectly, but not directly, related to both apology proclivity and apology effectiveness through difficulties with emotion regulation. The lack of a direct relationship between mothers’ childhood experiences of invalidation and apology characteristics suggests that maternal apology is linked to childhood experiences of invalidation only through the difficulties with emotion regulation that subsequently develops. The lack of a significant direct relationship could also be an artifact of our specific focus on apology as only one example of a validating parenting practice. However, similar research examining the direct and indirect associations between maternal history of childhood betrayal trauma and revictimization (an extreme form of the invalidating environment), maternal emotion regulation difficulties, and mothers’ negative responses to their children’s negative emotions, likewise found an indirect, but not direct, positive association between the mothers’ experiences of being invalidated and responding negatively to their children’s emotional experiences through the mothers’ emotion regulation difficulties [32]. Finally, had we measured mothers’ experience of apology from parents during childhood, we may have found a direct link. There may also potentially be a discrepancy between what mothers’ report they would say (while in a neutral emotional state completing a research study) and what they would ultimately do and say in an emotionally laden parenting situation.

These conclusions, however, should be tempered by the limitations of our cross-sectional and larger study design. Because we collected data at only a single timepoint, we were unable to examine mediation. Although we are interested in causal processes regarding parental invalidation and apology, and we have theoretical reasons to believe that retrospectively reported parental invalidation during childhood precede adult emotion regulation capacities, we cannot be conclusive about causality given the present study design. In addition, several other variables, such as parental empathy or warmth, child temperament, or parent–child attachment, may influence the relationship between childhood invalidation and maternal apology, and are unaccounted for by our model. These unmeasured variables should be investigated in future studies with longitudinal and experimental designs. Future studies may also benefit from examining more intense forms of invaliding environments that incorporate experiences of child abuse, as well as maternal apology strategies, where parents overcompensate and apologize too frequently, even for minor offenses.

Another limitation is the representativeness of our sample, which consisted of primarily White, middle-class, cisgender women from the Pacific Northwest. Future research should examine these relationships using more diverse ethnoracial and cultural samples, as parenting and apology are inherently influenced by cultural and environmental factors. Finally, future parenting research desperately needs to include fathers, and more specifically, racially/ethnically and culturally diverse fathers.

Despite these limitations, this is the first study to our knowledge to examine apology as a function of childhood experiences of invalidation or emotion regulation difficulties. It is unique in that it examines associations using two related, yet distinct, facets of apology among participants –individual proclivity to apologize to children and the apology quality when prompted to provide one – during a key developmental period for children’s socioemotional development [33]. Although both facets of apology operated similarly in the current study, and our measure of apology effectiveness was limited to one specific situation (see [17] for further discussion on the limitations of our analog measure), this initial research hopefully provides an important foundation for future investigations regarding both apology constructs.

The results of this study have meaningful implications. Mothers who experience difficulties regulating emotions, potentially because of their own childhood experiences of invalidation, may be less able or have a lower proclivity to apologize to their children. A better understanding of specific forms of parental invalidation, such as non-apology, is key to informing intervention efforts aimed at increasing parents’ abilities to help children develop healthy and adaptive emotion regulation skills. Lee and colleagues [11] recommend parent validation training to disrupt intergenerational cycles of parental invalidation, and we reiterate this suggestion with the addition of specific apology-related instruction. We recommend that parents, particularly those who may struggle with emotion regulation, be provided with skills to help regulate their emotions, as well as skills to recognize situations that may warrant an apology to their child and to craft an apology that is validating and effective.

## Conclusion

As a whole, this study provides preliminary evidence for direct and indirect links between parental apology, emotion regulation difficulties, and invalidating environments among a community sample of mothers. These results not only suggest the necessity of emotion regulation for the apology process, but also that parental apology specifically may be a useful parenting strategy to disrupt cycles of intergenerational transmission of invalidation and emotion dysregulation.

## Supplementary Information

**Additional file 1.**

## Data Availability

The datasets generated and/or analyzed during the current study are available in the Open Science Framework repository: https://osf.io/w74cy/?view_only=0b37483d24f046a7a70723149d3dce61

## Footnotes

1 This specific association has also been previously reported in [17], but it is provided in this paper for additional background.
