# Supplementary material for: Direct and indirect associations among mothers’ invalidating childhood environment, emotion regulation difficulties, and parental apology
Source: Borderline Personal Disord Emot Dysregul. 2022 Aug 18;9:21. doi: 10.1186/s40479-022-00191-z (PMC9387053; doi:10.1186/s40479-022-00191-z)
Supplement: Supplementary file 1 — Additional file 1. [file 40479_2022_191_MOESM1_ESM.docx]

Apology effectiveness was measured using mothers’ written responses to a hypothetical vignette. Mothers were asked to read the following vignette, authored by the study team, and to follow the instructions provided to script an apology for the scenario. They responded to the following text:

*Please read the following paragraph, describing a situation that a mom encounters in her daily life. You will be serving as a “coach” to this mom. When prompted, please provide the mom with the words that she should say to her child. A mom has been trying to get her 9-year old child to take better care of their possessions and to treat their possessions more responsibly. The mom arrives home after a long, stressful day. She sees that her child’s new bicycle is left outside, even after she had told her child to put it inside earlier that day. She confronts her child and begins yelling at them. She’s so angry that she doesn’t allow her child to say anything. Her child begins to cry. When the child is able to speak, the child tells the mom that the bike outside is not their bike. They did indeed put their bike away, and the child is not at fault. The mom feels guilty about overreacting and blaming her child, and she wants to apologize. Please write the exact words that the mom should say to her child verbatim (i.e., word-for-word, write as if the mom will use these words as a script when talking to her child). Please do not include any identifying information about you or your child*.

Coding manual for responses: https://mfr.osf.io/render?url=https://osf.io/c36dy/?pid=w74cy%26direct%26mode=render%26action=download%26mode=render.
